# Thoracic endometriosis syndrome: Cutting the gordian knot – A case report and review of the literature

**DOI:** 10.1016/j.ijscr.2019.11.032

**Published:** 2019-11-27

**Authors:** Oluwafolajimi A. Adesanya, Oludayo E. Kolawole

**Affiliations:** Cardiothoracic Surgery Unit, Department of Surgery, College of Medicine, University of Ibadan, Ibadan, Nigeria

**Keywords:** Thoracic Endometriosis Syndrome, Pleural effusion, Catamenial pneumothorax, Thoracotomy

## Abstract

**Introduction:**

Thoracic Endometriosis Syndrome (TES) is a rare disorder characterized by the presence of functional endometrial tissue in the thoracic cavity, often associated with the visceral or parietal pleura, lung parenchyma or airways. The aim of this case report is to illustrate the classic manner of presentation of TES and the modalities of therapy employed in a low-resource setting in the South-Western region of Nigeria.

**Case presentation:**

We report the case of a 37-year old woman who presented with right-sided pleuritic chest pain, cough and dyspnea closely associated with her menstrual cycle. On examination, there were reduced breath sounds and decreased air entry on the right hemithorax as well as dull percussion notes on the right middle and lower lung zones. Chest X-ray revealed signs of right-sided pleural effusion. Working diagnosis of right-sided Catamenial Pneumothorax was made for which she had 3 sessions of chemical pleurodesis which failed to close the air leaks. She eventually has a right-sided posterolateral thoracotomy and was co-managed by the O&G team with monthly Goserelin injections.

**Conclusion:**

This case illustrates the difficulties physicians in low-resource settings face in the diagnosis and treatment of TES and the importance of a high index of suspicion as well as a multidisciplinary approach in patient care.

## Highlights

• Thoracic Endometriosis Syndrome is a rare disorder.
• A strong relationship exists between thoracic endometriosis, pelvic endometriosis and infertility.
• Diagnosis remains a challenge particularly in low resource settings.
• Video-Assisted Thoracoscopic Surgery (VATS) remains the treatment of choice.

## 1 Introduction

Endometriosis is a pathology characterized by the presence of tissue histologically and functionally similar to the uterine endometrial tissue in places other than the lining of the uterine cavity [1]. While most anatomical locations for endometriosis are within the abdominopelvic cavity including: ovaries and fallopian tubes (96.4%), soft tissues (2.8%), gastrointestinal tract (0.3%) and urinary tract (0.2%) [2], a very small percentage occurs in the thorax. Thoracic Endometriosis Syndrome (TES) is a rare disorder characterized by the presence of functional endometrial tissue in the thoracic cavity, often associated with the visceral or parietal pleura, lung parenchyma or airways [3]. This condition presents in four different clinical forms – catamenial pneumothorax (73%), catamenial hemothorax (14%), catamenial hemoptysis (7%) and lung nodules (6%) [4]. A strong connection between thoracic endometriosis and pelvic endometriosis has been established as 50–84% of women with pelvic endometriosis have concomitant thoracic endometriosis [5]. TES is believed to be more common among the Caucasian and African American population [6], as not many cases in African women have been described. This is especially true for Nigeria, where a sparse number of cases have been diagnosed and even fewer reported; a situation attributable to a dearth of resources and expertise required for recognition, diagnosis and treatment of cases [6]. In line with the SCARE criteria, we report the case of a 37-year old woman with TES causing catamenial pneumothorax [7].

## 2 Case report

**Fig. 1:**
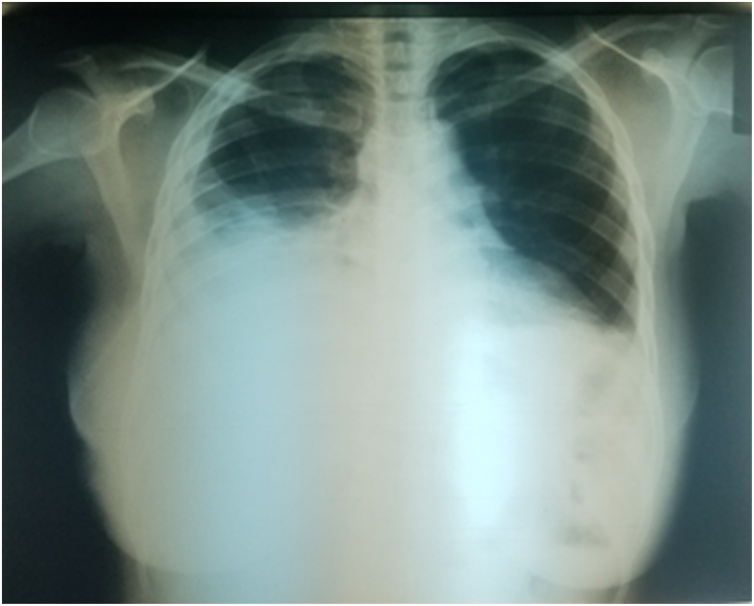
Chest X-ray at presentation showing massive right-sided pleural effusion.

**Fig. 2:**
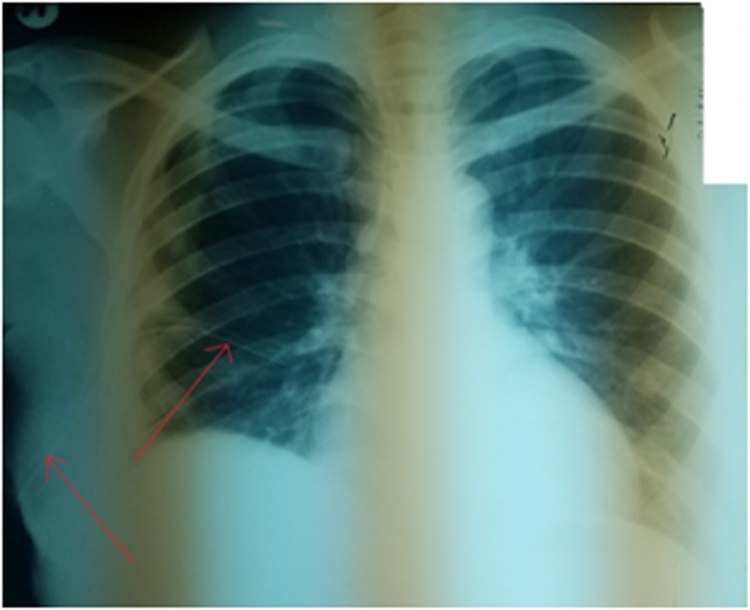
Chest X-ray showing resolving right-sided pleural effusion post Thoracostomy. Arrows show chest tube in situ.

A 37-year old nulliparous African female presented to the Emergency Department with recurrent right-sided chest pain, cough and progressive dyspnoea of 2 months duration, co-inciding with the onset of menstruation. She had previously been managed with Closed Thoracostomy Tube Drainage (CTTD). She was being managed by the Obstetrics & Gynaecology (O&G) unit for secondary infertility and severe dysmenorrhoea. She neither smokes nor takes alcohol and her family history was unremarkable. On examination she had decreased air entry in right hemithorax, stony dull percussion notes and absent breath sounds on the right lower and middle lung zones. Examination of other systems was unremarkable. Vital signs on presentation: respiratory rate (30 cycles/minute), temperature (36.7 °C), pulse rate (90 beats/minute), blood pressure (100/70 mmHg), Sp0_2_ in room air (97%) and pain score of 8/10 as evaluated by the Visual Analog Scale of 1–10 [1 = no pain; 10 = agonizing pain]. Chest X-ray (Fig. 1) revealed massive right-sided pleural effusion with pneumothorax involving the middle and lower lung zones. Ultrasonography of the pleura revealed a 3.2 × 1.4 cm anechoic right-sided pleural collection anteriorly at the mid-clavicular line, just above the ipsilateral diaphragm. She had a diagnostic thoracocentesis which revealed aspirate of air and frankly hemorrhagic effluent, necessitating her admission by the Cardiothoracic Surgery Unit with a working diagnosis of right-sided TES with a suspicion of catamenial pneumothorax and catamenial hemothorax. On Admission, she had a right-sided CTTD (Fig. 2), which drained an initial volume of 1.1 litres of hemorrhagic effluent, and a pleural biopsy. Pleural biopsy report revealed widespread inflammation in the pleura, a finding supported by her Full Blood Count (FBC) panel, which revealed Leukocytosis (13,400 cells/mm^3^) with Neutrophilia of 82%. She had a serum CA-125 level of 55.5 u/ml. Over the following weeks, she had 4 repeat CTTD procedures due to persistent drainage of frothy, hemorrhagic effluent and persistent alveolopleural fistulas (air leaks) as shown by her repeat X-ray films. She also had 2 chemical pleurodesis procedures which failed to close the air leak.

**Fig. 3:**
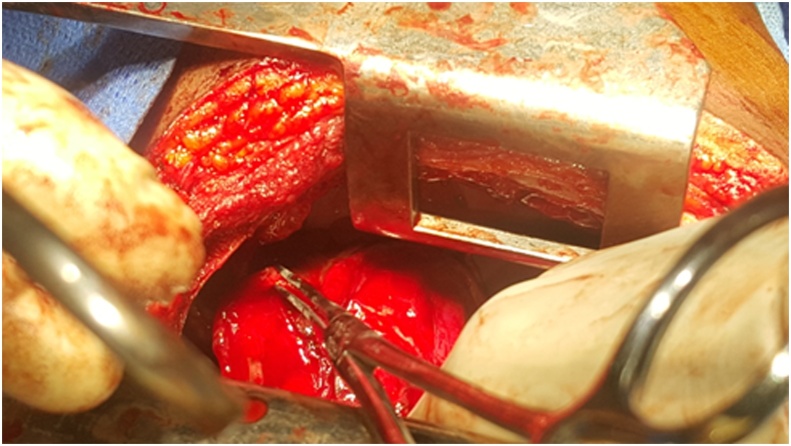
Intra-operative image showing alveolopleural fistula identification and closure.

The patient was eventually scheduled for right-sided posterolateral thoracotomy with repair of alveolopleural fistula and mechanical pleurodesis. Alveolopleural fistulas were identified and closed using 2-0 Vicryl sutures (Fig. 3). Mechanical pleurodesis was done by abrasion of the pleura using sterile gauze. Ribs were re-approximated using 2 Vicryl sutures, muscle with 1 Vicryl suture, subcutaneous tissue with 2-0 Vicryl suture and skin with 3-0 Monocryl suture. The immediate post-op status was stable with optimal pain control. She had a repeat Chest X-ray on the 6th day post-op which showed an improved status and she was subsequently discharged. She was co-managed by the O&G team with monthly Goserelin injections and tabs mefenamic acid and hematinics. The procedure was done under General Anesthesia by a Cardiothoracic Surgical Senior Resident with over 3 years of specialty training.

## 3 Discussion

Thoracic Endometriosis Syndrome (TES) is a rare disorder characterized by the presence of functional endometrial tissue in the pleura, lung parenchyma and airways [3]. It affects 5–15% of women in their reproductive years [8], with the mean age at presentation being 35 ± 0.6 years and a range from 15 to 54 years [5]. Meanwhile 92% of TES cases occur in the right hemithorax [involving mainly the pleura and less often the lung parenchyma itself], 5% in the left hemithorax and 3% bilateral involvement [9]. In our case, the patient falls within the age range and had a right hemithoracic involvement.

Many factors have been described to influence the risk of developing TES (Table 1). While early age at menarche, nulliparity and short menstrual cycle increase the risk due to increased exposure to high levels of circulating estradiol which promotes endometrial tissue development, high body mass index (BMI) is associated with decreased risk [10]. In our case, the patient was nulliparous. Other listed factors have a mixed association with risk of TES.

**Table 1:** Factors influencing risk of developing endometriosis [10].

Factors associated with increased risk	Factors associated with decreased risk
Early age at menarche	Parity
Shorter menstrual cycle	Current oral contraceptive use
Taller height	Smoking
Alcohol use	Higher body mass index
Caffeine intake	Regular exercise

A universal consensus is yet to be reached on the etiology and pathogenesis of TES but many theories have been proposed. Coelomic Metaplasia explains a pathologic stimulus could induce mesothelial cells to differentiate into endometrial cells due to the common embryonic origin of both cell lines [8]. The migration theory suggests the migration of endometrial tissue implants from the uterus through the fallopian tubes into the pelvic cavity [11]. Micro-embolization theory explains the lymphogenous spread of endometrial tissue emboli from the uterine cavity to the lungs [11]. Finally, Sampson’s theory - which states that retrograde menstruation causes intraperitoneal spilling of endometrial cells and subsequent adhesion to the peritoneal surface [12].

TES presents as any of 4 distinct clinical entities: catamenial pneumothorax (CP), catamenial hemothorax (CH), catamenial hemoptysis (CHt) and lung nodules [4,5]. Common symptoms on presentation include: severe pleuritic chest pain [often right-sided, peri-scapular and radiating to the neck], dyspnea and cough [8,13,14]. In our case, the patient had all three symptoms.

Diagnosis of TES is often challenging. A typical history will be that of a woman in her reproductive years presenting with chest pain, dyspnea and cough around the time of her menstruation. On examination, there would be decreased/absent breath sounds and reduced chest expansion on the affected side [9]. More than 60% of affected patients may require a thoracotomy/thoracoscopy at presentation for successful diagnosis [3]. Chest roentgenogram shows signs of pleural effusion, pneumothorax and less commonly, pulmonary nodules. Computerized Tomography (CT) scan reveals endometrial implants as hypo-attenuating areas on the pleura and the lung parenchyma [14]. Endometrial implants on MRI will appear as hyper-intense areas. [14]. Abdominal Ultrasonography is also important because most cases of TES have concomitant pelvic endometriosis [5]. Histological diagnosis with pleural or lung biopsy reveals hemosiderin-laden macrophages; however, histological diagnosis is obtained in 1/3 of available reported cases in literature, suggesting its insufficiency in diagnosis [3,4]. Histological diagnosis was not obtained in our patient. Currently, there is no reliable biomarker in the endometrial tissue, uterus, blood or urine for diagnosis of endometriosis [10], however, serum CA-125 level is the closest to one. The optimum cut-off level of CA-125 in diagnosis is 39 u/ml. Our patient had a CA-125 level of 55.5 u/ml at presentation. The disadvantage of CA-125 is that its levels in the serum or pleura fluid can be increased by any irritation to the mesothelial cells [15]. The gold standard for the diagnosis remains video-assisted thoracoscopic surgery (VATS) because it allows for the direct visualization of the lung and pleural surfaces for endometrial tissue implants [10,14,15].

Existing treatment modalities are divided into medical, surgical and combined therapy [3,17,19]. The goal of medical (or hormonal) therapy is suppression of ovarian estrogen secretion [1,14], using oral contraceptives, progesterone agonists, gonadotropin-releasing hormone agonists (GnRH) and danazol. Our patient was being co-managed by the O&G team with Goserelin, a GnRH agonist. The gold-standard for surgical treatment of TES is video-assisted Thoracoscopic surgery (VATS) as it allows for a broad range of surgical treatment modalities including: closure of diaphragmatic defects, mechanical pleurodesis, chemical pleurodesis and lung resection [16]. In many low resource settings, VATS may be unavailable, and open thoracotomy should be employed. Treatment with chemical pleurodesis alone is associated with a very high rate of recurrence due to continuous migration of endometrial tissue through diaphragmatic defects into the thorax when such defects are not closed [3,15,18]. While surgical therapy has been found to be more effective at preventing recurrence than hormonal therapy [16], combination of both surgical and hormonal therapies have been associated with no recurrence in a follow up period of up to 45 months [14].

## 4 Conclusion

TES is an extremely rare condition, the diagnosis of which is usually based on exclusion. Our patient presented in the ‘typical’ manner with chest pain, dyspnea and cough closely related to menstruation. Management of TES usually involves the combined efforts of the cardiothoracic surgeons, gynecologists and chest physicians and although the gold standard for surgical therapy was not available, the patient was successfully treated with a combination therapy of conventional open thoracotomy and hormonal therapy.

## Ethical approval

We have the consent of the patient. We have not submitted the case to the Ethics Committee.

## Consent

Written informed consent was obtained from the patient for publication of this case report and accompanying images. A copy of the written consent is available for review by the Editor-in-Chief of this journal on request.

## Author contribution

Oluwafolajimi A. Adesanya: Study Concept or Design, Data Curation, Literature Review, Writing - Original Draft.

Oludayo E. Kolawole: Writing - Review & Editing.

## Registration of research studies

As this is a Case Report and not a Clinical Trial or a First in Man research, this study does not require registration.

## Guarantor

Oluwafolajimi A. Adesanya.

## Provenance and peer review

Not commissioned, externally peer-reviewed.

The author(s) declare that they have no conflicts of interest.

## Funding

This research did not receive any specific grant from funding agencies in the public, commercial, or not-for-profit sectors.

## Declaration of Competing Interest

The author(s) declare that they have no conflicts of interest.
